# Molecular Characterization of Human Respiratory Syncytial Virus in the Philippines, 2012-2013

**DOI:** 10.1371/journal.pone.0142192

**Published:** 2015-11-05

**Authors:** Rungnapa Malasao, Michiko Okamoto, Natthawan Chaimongkol, Tadatsugu Imamura, Kentaro Tohma, Isolde Dapat, Clyde Dapat, Akira Suzuki, Mayuko Saito, Mariko Saito, Raita Tamaki, Gay Anne Granada Pedrera-Rico, Rapunzel Aniceto, Reynaldo Frederick Negosa Quicho, Edelwisa Segubre-Mercado, Socorro Lupisan, Hitoshi Oshitani

**Affiliations:** 1 Tohoku University Graduate School of Medicine, Sendai, Japan; 2 Tohoku-RITM Collaborating Research Center on Emerging and Reemerging Diseases, Muntinlupa City, Philippines; 3 Biliran Provincial Hospital, Naval City, Philippines; 4 Eastern Visayas Regional Medical Center, Tacloban City, Philippines; 5 Ospital ng Palawan, Puerto Princesa City, Philippines; 6 Research Institute for Tropical Medicine, Muntinlupa City, Philippines; University of Melbourne, AUSTRALIA

## Abstract

Human respiratory syncytial virus (HRSV) is a major cause of acute lower respiratory tract infections in infants and children worldwide. We performed molecular analysis of HRSV among infants and children with clinical diagnosis of severe pneumonia in four study sites in the Philippines, including Biliran, Leyte, Palawan, and Metro Manila from June 2012 to July 2013. Nasopharyngeal swabs were collected and screened for HRSV using real-time polymerase chain reaction (PCR). Positive samples were tested by conventional PCR and sequenced for the second hypervariable region (2^nd^ HVR) of the G gene. Among a total of 1,505 samples, 423 samples were positive for HRSV (28.1%), of which 305 (72.1%) and 118 (27.9%) were identified as HRSV-A and HRSV-B, respectively. Two genotypes of HRSV-A, NA1 and ON1, were identified during the study period. The novel ON1 genotype with a 72-nucleotide duplication in 2^nd^ HVR of the G gene increased rapidly and finally became the predominant genotype in 2013 with an evolutionary rate higher than the NA1 genotype. Moreover, in the ON1 genotype, we found positive selection at amino acid position 274 (p<0.05) and massive O- and N-glycosylation in the 2^nd^ HVR of the G gene. Among HRSV-B, BA9 was the predominant genotype circulating in the Philippines. However, two sporadic cases of GB2 genotype were found, which might share a common ancestor with other Asian strains. These findings suggest that HRSV is an important cause of severe acute respiratory infection among children in the Philippines and revealed the emergence and subsequent predominance of the ON1 genotype and the sporadic detection of the GB2 genotype. Both genotypes were detected for the first time in the Philippines.

## Introduction

Acute lower respiratory tract infection (ALRI) is a severe disease that leads to the morbidity and mortality in infants and children worldwide. One of the most common causes of ALRI is human respiratory syncytial virus (HRSV). Globally, 2.8 to 4.3 million children are admitted to hospitals and approximately 66,000 to 199,000 children infected with HRSV aged less than 5 years old die annually, particularly in developing countries [1]. HRSV is a member of family *Paramyxoviridae*, genus *Pneumovirus*. The HRSV virion is 100–350 nm in diameter. It has an envelope and a linear, negative-sense, and single-stranded RNA genome of approximately 15.2 kb with helical nucleocapsid. The genome has 10 genes, which encodes 11 proteins. The G gene encodes for the G protein, a type II glycoprotein on the virus envelope involved in attachment during virus entry. The G protein, which is heavily glycosylated with N-linked and, especially, O-linked sugars [2], contains two hypervariable regions (HVR), the 1^st^ HVR and 2^nd^ HVR, flanking a central conserved region.

HRSV is classified into 2 subgroups, HRSV-A and HRSV-B, based initially on their antigenic differences [2]. The prototype HRSV-A strain, A2, was isolated in Australia in 1961 while the prototype HRSV-B strain, SW8/60, was detected in Sweden in 1960 [3]. At present, both HRSV subgroups are classified further into genotypes based on the sequence variability of the 2^nd^ HVR of the G gene. HRSV-A can be divided into 11 genotypes: GA1-GA7, SAA1, NA1-NA2 and the 72-nucleotide duplication genotype, ON1. HRSV-B can be divided into 9 genotypes: GB1-GB4, SAB1-SAB4 and the 60-nucleotide duplication genotype, BA, which has 12 minor groups (BA1-12) [4–9].

Insertions and deletions in the G protein of HRSV have been reported previously [3, 10, 11]. A noteworthy G gene modification in HRSV-B, a 60-nucleotide duplication in the second hypervariable region, was detected in 1999 in Buenos Aires, Argentina [12]. This new genotype, named BA, has become predominant worldwide and has even replaced previously circulating HRSV-B strains in most countries [12]. However, in recent years, a reemergence of non-BA genotypes has been observed [13]. Another notable G gene change within the same region, a 72-nucleotide duplication in HRSV-A, was identified in 2010 in Ontario, Canada [4]. This new genotype, named ON1, was subsequently found in Germany, Italy, Croatia, Latvia, South Africa, and Kenya [4, 14–18]. In Asia, the ON1 genotype has been detected in Malaysia, South Korea, China, India, Thailand, and Japan [8, 19–24].

The etiological significance of HRSV has been shown in many countries worldwide, including in developing countries. In the Philippines, a previous study conducted in 2008–2009 revealed that HRSV was the second most common virus (24.1%) among hospitalized children with severe pneumonia, next to rhinoviruses [25]. In our previous study from 2008 to 2012, HRSV was detected in 415 children admitted to hospitals with severe pneumonia. Almost half of the HRSVs were identified as HRSV-A, and all of them were genotype NA1. The remaining HRSVs were HRSV-B genotype BA9 [7]. In this study, we aim mainly to genetically characterize HRSV strains circulating in the Philippines between June 2012 and July 2013. To infer the evolutionary dynamics of Philippine HRSVs, strains detected from our previous study from May 2008 to May 2012 were included in the analysis.

## Materials and Methods

### Ethics statement

This study was approved by the Biliran Provincial Hospital (BPH), the Eastern Visayas Regional Medical Center (EVRMC), the Ospital ng Palawan (ONP), and the Institutional Review Boards of the Research Institute for Tropical Medicine (RITM) as well as the Ethical Committee of Tohoku University Graduate School of Medicine. Written and verbal informed consents were obtained from parents or guardians prior to enrollment of children in this study.

### Clinical specimens

A total of 1,505 nasopharyngeal swabs (NPS) were collected from children aged less than 14 years who sought hospital care or referred to the hospital with clinical diagnosis of severe pneumonia based on the Integrated Management of Childhood Illness (IMCI) guidelines [26] from June 2012 to July 2013 from four study sites in the Philippines, including 549 samples from the Eastern Visayas Regional Medical Center (EVRMC) in Tacloban City of Leyte Island, 456 from the Ospital Ng Palawan (ONP) in Puerto Princesa City of Palawan Island, 431 from the Biliran Provincial Hospital (BPH) in Naval City of Biliran Island, and 69 from the Research Institute for Tropical Medicine (RITM) in Metro Manila. All specimens were placed in viral transport medium (VTM) and transported to RITM with ice packs for further analysis.

### Viral RNA extraction, cDNA synthesis, Polymerase Chain Reaction (PCR) and Sequencing

After centrifugation, the supernatant was collected and viral RNA was extracted using QIAamp^®^ MinElute^®^ Virus Spin kit. (Qiagen, Hilden, Germany). The viral RNA was reverse-transcribed to complementary DNA (cDNA) using Moloney Murine Leukemia Virus (M-MLV) reverse transcriptase and random primers (Invitrogen Carlsbad, CA, USA). Real-time PCR was performed for HRSV screening [27] and the primers used for this study are shown in S1 Table. HRSV positive samples were further subtyped into HRSV-A or HRSV-B by hemi-nested PCR as described previously [7]. PCR products were purified using QIAquick^®^ PCR Purification Kit (Qiagen, Hilden, Germany) and then nucleotide sequencing by Sanger dideoxy technique was performed using Big Dye Terminator version 1.1 cycle sequencing kit and Genetic Analyzer 3730 (Applied Biosystems, Foster City, USA).

Representative nucleotide sequences, which were considered to be unique sequences of HRSV-A (accession number KM873377—KM873459) and HRSV-B (accession number KM873460—KM873510) were submitted to GenBank.

### Phylogenetic analysis

To infer the genetic relationship among viruses, maximum-likelihood analysis was performed using MEGA5.2 software [28]. Phylogenetic trees of Philippine sequences of HRSV-A (NA1 and ON1) and HRSV-B (GB2 and BA9) were constructed. BLASTn search results of the nearest relatives of Philippine HRSV strains were included in the analysis. Statistical support was evaluated using bootstrap method with 1,000 replicates and bootstrap values ≥70% are shown on the branches of the consensus tree [29].

### Analysis of deduced amino acid sequence, selective pressure, and prediction of N- and O-glycosylations

The selective pressure of deduced amino acid sequences of each genotype was evaluated using the estimate selection for each codon method in Hyphy program [30], which is available in MEGA 5.2 software [28]. If the ratio of the proportion of nonsynonymous substitutions to synonymous substitutions, dN/dS, is > 0, = 0, or < 0, this would indicate that the site is under positive, neutral, or negative selection, respectively. Amino acid sequences of Philippine strains were compared with the prototype sequence of genotype NA1 (NG-016-04, accession number: AB470478) [5]; ON1 (ON67-1210A, accession number: JN257693) [4]; GB2 (CH93-9b, accession number: AF065251) [31], and BA (BA3833/99B, accession number: AY333362) [32]. These prototype strains are early reported strains that represent the genotype and are used as basis for comparison of newer strains.

To predict potential O-linked and N-linked glycosylation sites of the 2^nd^ HVR of the G protein, NetOGlyc 4.0 and NetNGlyc 1.0 (http://www.cbs.dtu.dk/services/NetOGlyc/ and http://www.cbs.dtu.dk/services/NetNGlyc/) were used. O-linked glycosylation is based on amino acids serine (Ser) and threonine (Thr) and N-linked glycosylation is based on the amino acid configuration Asn-Xaa-Ser/Thr except proline (Pro).

### Entropy analysis

To evaluate the amino acid variability across the 2^nd^ HVR of the G gene, Shannon entropy analysis as implemented in the BioEdit software was performed [33, 34]. The entropy plot is a measure of variability at a particular amino acid in the alignment. The entropy values were exported and plotted in Microsoft Excel. In this analysis, the range of the Shannon entropy values was from 0 to 0.8 with the Shannon entropy threshold value set to 0.2, which was determined from the highest entropy value representing amino acid residues in the conserved region (amino acid position 164–186 of G protein). Amino acids with entropy value <0.2 are considered conserved. On the other hand, amino acids with entropy value >0.2 are considered variable.

### Phylodynamic analysis

To determine the evolutionary dynamics of HRSV-A and HRSV-B in the Philippines, Bayesian Markov Chain Monte Carlo (MCMC) method was used as implemented in the BEAST V.1.8.0 software package [35]. The datasets include only sequence data of genotypes NA1, ON1, and BA9 Philippine HRSVs. Sequences of Philippine HRSVs collected between June 2012 and July 2013, as well as sequences from our previous study collected between May 2008 to April 2012 were included in the analysis [7]. The best-fitting nucleotide substitution model on each dataset, which was selected using MEGA 5.2 software, was used in the analysis. To estimate the rate of mutation and time to most recent common ancestor (TMRCA), an uncorrelated relaxed clock model was selected. To estimate the genetic diversity of population over time, a skyline plot analysis was performed. For each dataset, 200 million MCMC chains and sampling every 20,000 generations were performed. To calibrate the molecular clock, prior evolutionary rates obtained from the published papers including 3.57 x 10^−3^ (95% HPD, 2.44 x 10^−3^–4.65 x 10^−3^) substitutions/site/year for HRSV-A [24] and 5.89 x 10^−3^ (95% HPD, 1.43 x 10^−3^–7.63 x 10^−3^) substitutions/site/year for HRSV-B [17] were used in the analysis. Convergence was assessed based on the effective sample size (ESS) value >200 of each parameter on every run using TRACER V.1.5 software [35]. The best-fitting model of each dataset was selected based on the lowest Akaike’s information criterion through MCMC (AICM) value. The maximum clade credibility (MCC) tree was inferred using TreeAnnotator v.1.8.0 and visualized using FigTree v.1.4.0 software. To estimate the uncertainty in the year for each node, the 95% highest posterior density (HPD) intervals are indicated on the node bars.

To infer the evolutionary relationship of Philippine GB2 strains with strains from other countries, sequences of two GB2 strains detected in the Philippines were used as queries for BLASTn search (http://blast.ncbi.nlm.nih.gov/Blast.cgi?CMD=Web&PAGE_TYPE=BlastHome). The top 100 results and previously reported GB2 strains (BE/90/92, accession number AY751247) [10] were included in the analysis. About 100 million MCMC chains and sampling every 10,000 generations were performed. Assessment of convergence and visualization of MCC tree were performed as described above.

As an independent estimate of the temporal signal of sequence data, root-to-tip linear regression analysis was performed as implemented in the Path-O-Gen program (tree.bio.ed.ac.uk/software/pathogen). The software assesses how genetic distance correlates with sampling date.

## Results

### Prevalence of HRSV from 2012–2013

In four study sites in the Philippines from 2012–2013, 423 (28.1%) out of 1,505 nasopharyngeal swabs were positive for HRSV. Among the total specimens collected, 94.8% were from severe and neonatal pneumonia cases. In each study site, 24.8% (136/549) were positive for HRSV from the EVRMC in Tacloban City of Leyte Island, 30.5% (139/456) were positive for HRSV from the ONP in Puerto Princesa City of Palawan Island, 30.6% (132/431) were positive for HRSV from the BPH in Naval City of Biliran Island, and 23.2% (16/69) were positive for HRSV from the RITM in Metro Manila.

Among the HRSV-positive cases, 305 (72.1%) were HRSV-A and 118 (27.9%) were HRSV-B. Among the HRSV-A cases, 204 (66.9%) were ON1 genotype and 101 (33.1%) were NA1 genotype while for the HRSV-B cases, 116 (98.3%) were BA9 genotype and 2 (1.7%) were GB2 genotype.

**Fig 1:**
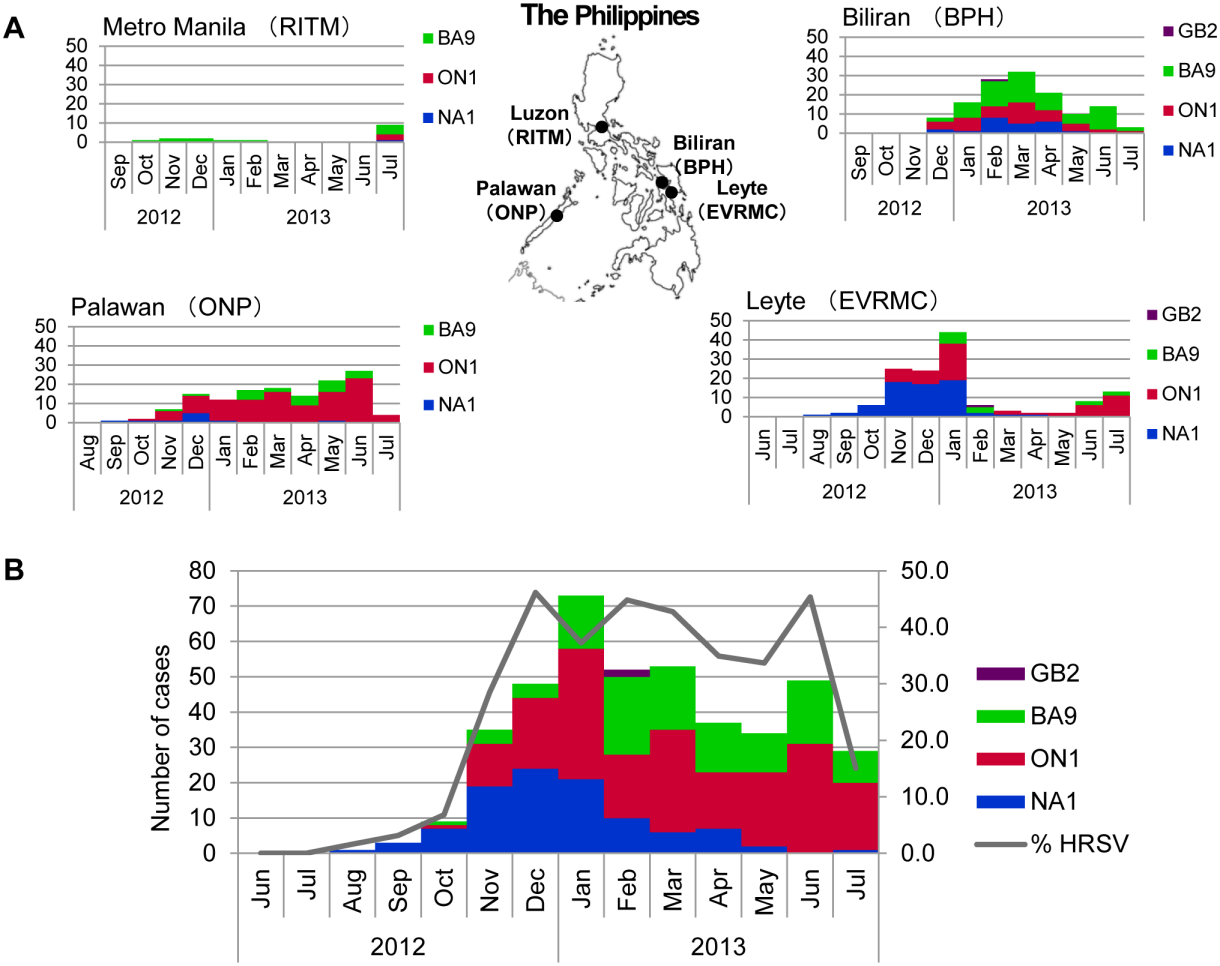
Temporal distribution of HRSV strains detected in 2012–2013 in the Philippines. (A) Monthly distribution of HRSV cases in each study site including BPH in Naval City of Biliran Island, EVRMC in Tacloban City of Leyte Island, ONP in Puerto Princesa City of Palawan Island, and RITM in Metro Manila according to genotype. (B) Overall temporal distribution of HRSV strains in four study sites from the period June 2012 to July 2013 in the Philippines.

Temporal distribution of genotypes in the four study sites is shown in Fig 1A. The novel HRSV-A ON1 genotype with a 72-nucleotide duplication was detected for the first time in Palawan, an island located separately from the main group of islands. Moreover, the number of ON1 cases in Palawan (105 cases) was twice that in Leyte (55 cases) or Biliran (41 cases). Notably, sporadic cases of GB2, a non-BA HRSV-B genotype, were detected in Leyte and Biliran.

The peak of HRSV activity occurred in January 2013 (Fig 1B). Among HRSV-A genotypes, NA1 was first detected in August 2012 and continued to circulate until July 2013. Genotype ON1 was first detected in October 2012, rapidly increased and became the dominant genotype in 2013. Among HRSV-B genotypes, BA9 was the predominant strain while GB2 was only detected in February 2013.

### Phylogenetic analysis of HRSV

**Fig 2:**
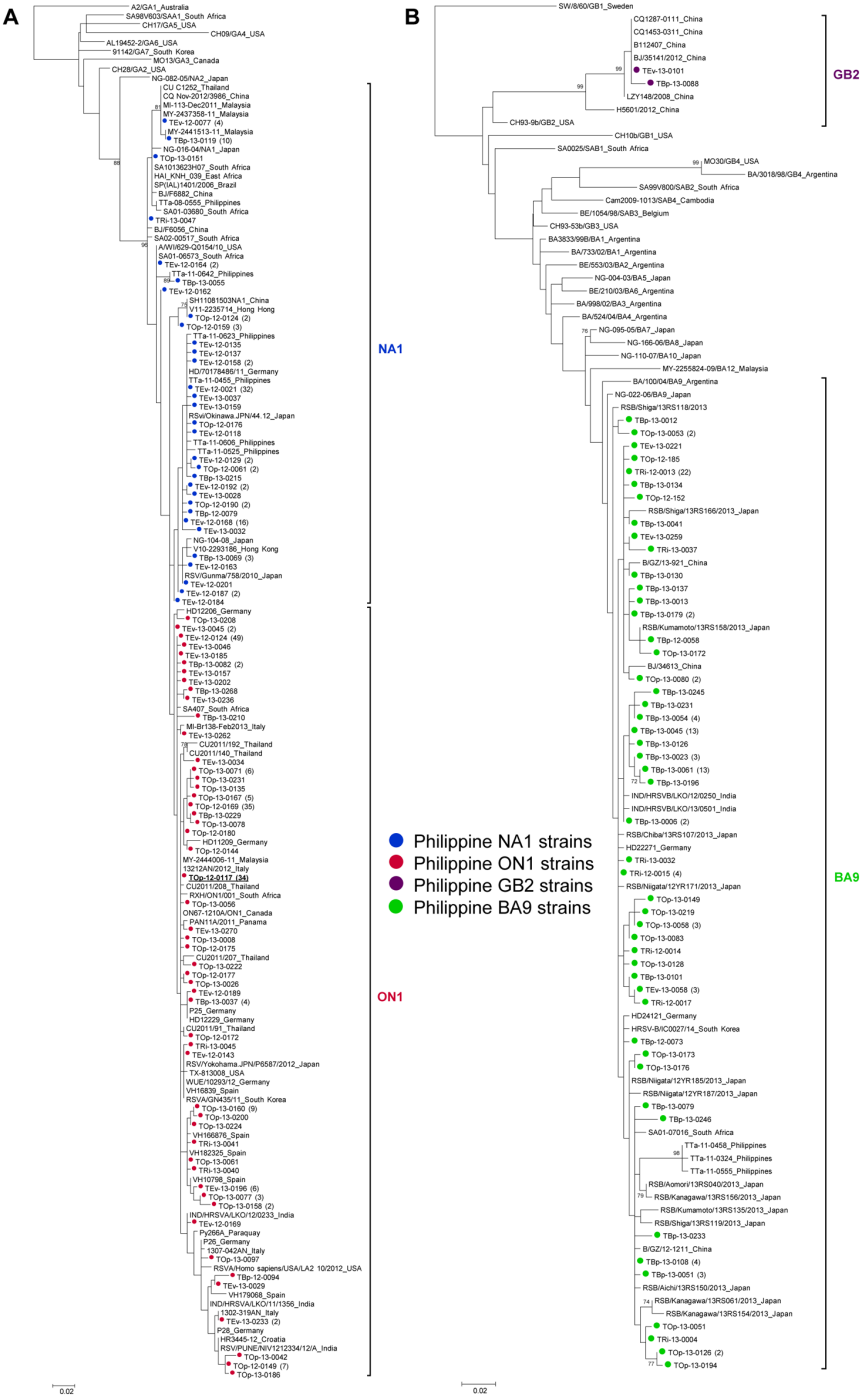
Phylogenetic analysis of representative HRSV in epidemic season 2012/13. (A) Phylogenetic tree of HRSV-A based on the nucleotide sequences of the second hypervariable region (2^nd^ HVR) of the G gene; 342 nucleotides for the 72-nucleotide duplication genotype (ON1 genotype) and 270 nucleotides for the non-duplication genotypes (GA1-7, SAA1, NA1-2 genotypes). (B) Phylogenetic tree of HRSV-B based on the nucleotide sequences of the 2nd HVR of the G gene; 330 nucleotides for the 60-nucleotide duplication genotype (BA genotype) and 270 nucleotides for the non-duplication genotypes (GB1-4, SAB1-4 genotypes). Bootstrap values >70 derived from 1,000 bootstrap replications for evaluating the confidence estimates are shown at branch nodes. The parameters, “all sites” for gap or missing data treatment and “very strong” for branch swap filter, were used in this study. The trees were constructed by adding strains from other countries that were identified by BLASTn search. Genotypes of reference strains from previous studies [4, 36] are indicated in the strain names. Representative strains detected in this study are indicated by the following: NA1 strains (blue circles); ON1 strains (red circles); GB2 strains (purple circles); BA9 strains (green circles). The first ON1 strain that was detected in the Philippines is boldfaced and underlined. Figures inside the parenthesis represent the number of identical sequences.

The 2012–2013 HRSV-A strains were distributed into two clusters, NA1 and ON1 (Fig 2A). The phylogenetic tree was constructed by including strains from other countries that were identified by BLASTn search. The topology of the tree showed no paraphyly of Philippine strains. The Philippine NA1 strains were closely related to the previously circulating strains in the country in 2008 and 2011, and strains from other countries. The Philippine ON1 strains also clustered with strains detected in other countries including Canada, USA, Panama, Paraguay, South Korea, Japan, Thailand, Malaysia, India, Germany, Spain, Italy, Croatia, and South Africa.

All 118 HRSV-B strains were identified as BA9 genotype except for 2 strains, which were identified as GB2 genotype (Fig 2B). The tree was generated by adding strains from other countries that were identified by BLASTn search. All BA9 strains clustered with strains from other countries while the two Philippine GB2 strains clustered with Chinese strains.

Pairwise distance analysis showed that the average pairwise distances within the ON1 genotype and within the NA1 genotype were 0.017 and 0.016, respectively, while the mean distance between the two genotypes was 0.033. The average pairwise distances within the BA9 genotype and within the GB2 genotype were 0.016 and 0.007, respectively. The mean distance between the two HRSV-B genotypes was 0.114.

### Genetic diversity in the 2^nd^ HVR of the G gene

**Fig 3:**
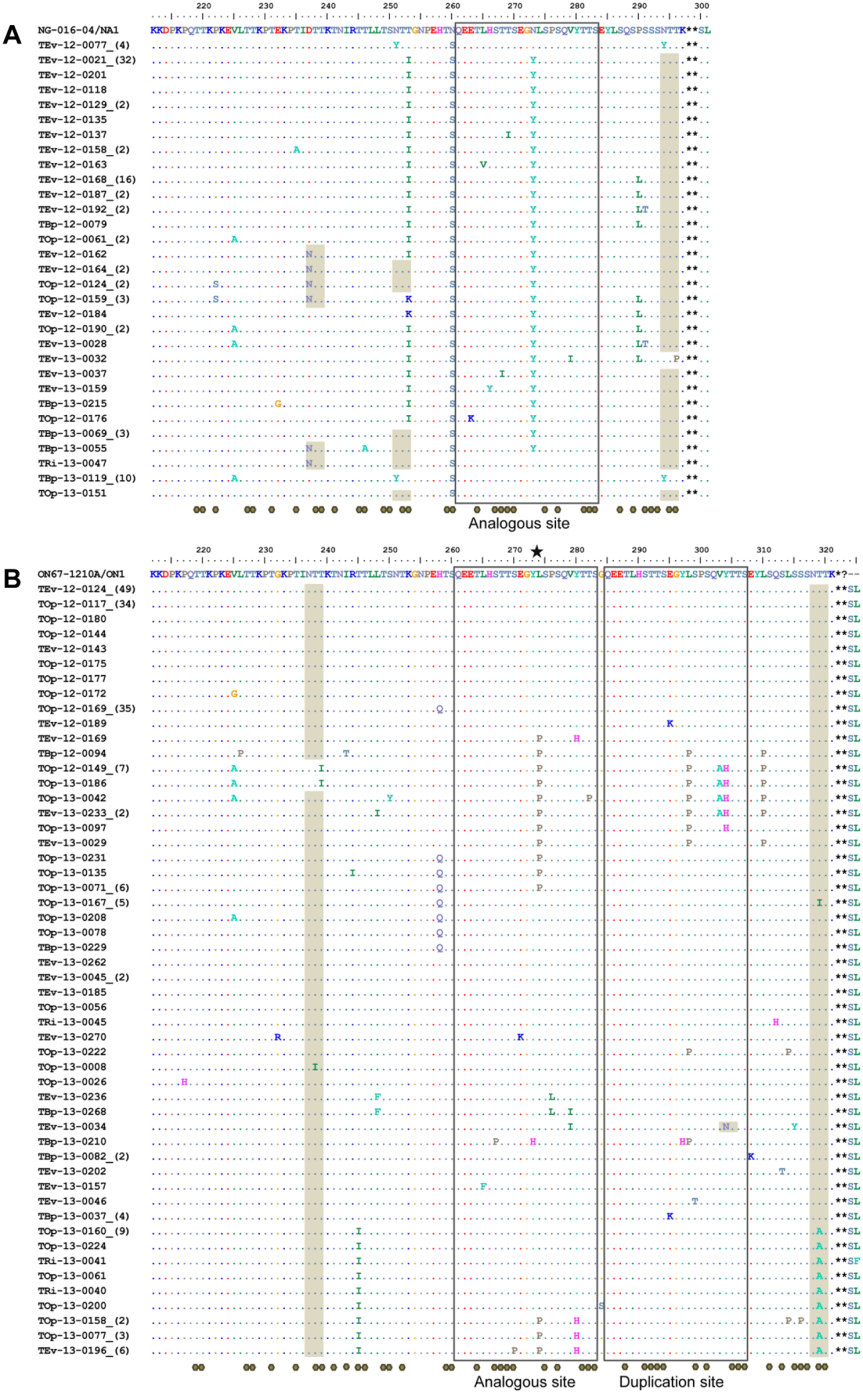
Deduced amino acids of representative HRSV-A G protein in 2012–2013. (A) Thirty-one representative unique NA1 amino acid sequences corresponding to position 212 to 301 of the 2^nd^ HVR of G protein were aligned with the prototype NA1 strain, NG-016-04 (AB470478). (B) Fifty-two representative unique ON1 amino acid sequences corresponding to position 212 to 325 of the 2nd HVR of G protein were aligned with the prototype ON1 strain, ON67-1210A (JN257693). Gray shading for predicted N-linked sites, gray octagons for predicted O-linked sites, dots for identical residues, asterisks for stop codon positions, star for positive selection position, boxes for analogous sites/duplication sites, and parenthesis for number of identical strains are indicated.

The G protein is highly variable and heavily glycosylated with N- and O-linked sugars. To investigate the diversity in the 2^nd^ HVR of G protein, representative HRSV strains with unique sequences from the Philippines from 2012–2013 were compared with the prototype strains for genotype NA1 (NG-016-04), ON1 (ON67-1210A), GB2 (CH93-9b), and BA (BA3833/99B). Compared with the NG-016-04 strain, all NA1 strains from the Philippines had N260S amino acid substitution and most had T253I and N273Y amino acid substitutions in the analogous site, the site that corresponds to the duplicated region of the G gene (Fig 3A). The predicted N-linked and O-linked glycosylation were found in 3 and 36 sites, respectively. Forty-three percent (88/204) of the 2012–2013 Philippine ON1 strains had identical deduced amino acid sequence in the 2^nd^ HVR of the G protein as the prototype Canadian strain, ON67-1210A (Fig 3B). Within the 23-amino acid duplication sites, lysine (L) to proline (P) and tyrosine (Y) to histidine (H) substitutions were found in the exact same positions in both sites in several ON1 strains. An additional valine (V) to alanine (A) amino acid change was found in the duplicated site in 11 strains. Outside of the duplication sites, V225A, T245I, H258Q and L310P substitutions were observed in some ON1 strains. N-linked and O-linked glycosylation was predicted in 2 and 45 sites, respectively, which also included the predicted O-linked sugars in the duplication sites. Amino acid mutations within the N-linked glycosylation sites such as Tyr to Ile at position 239 led to a loss of glycosylation while the change Tyr to Ala at position 319 did not. Moreover, amino acid position 274 in the analogous site was found to be positively selected (p<0.05).

**Fig 4:**
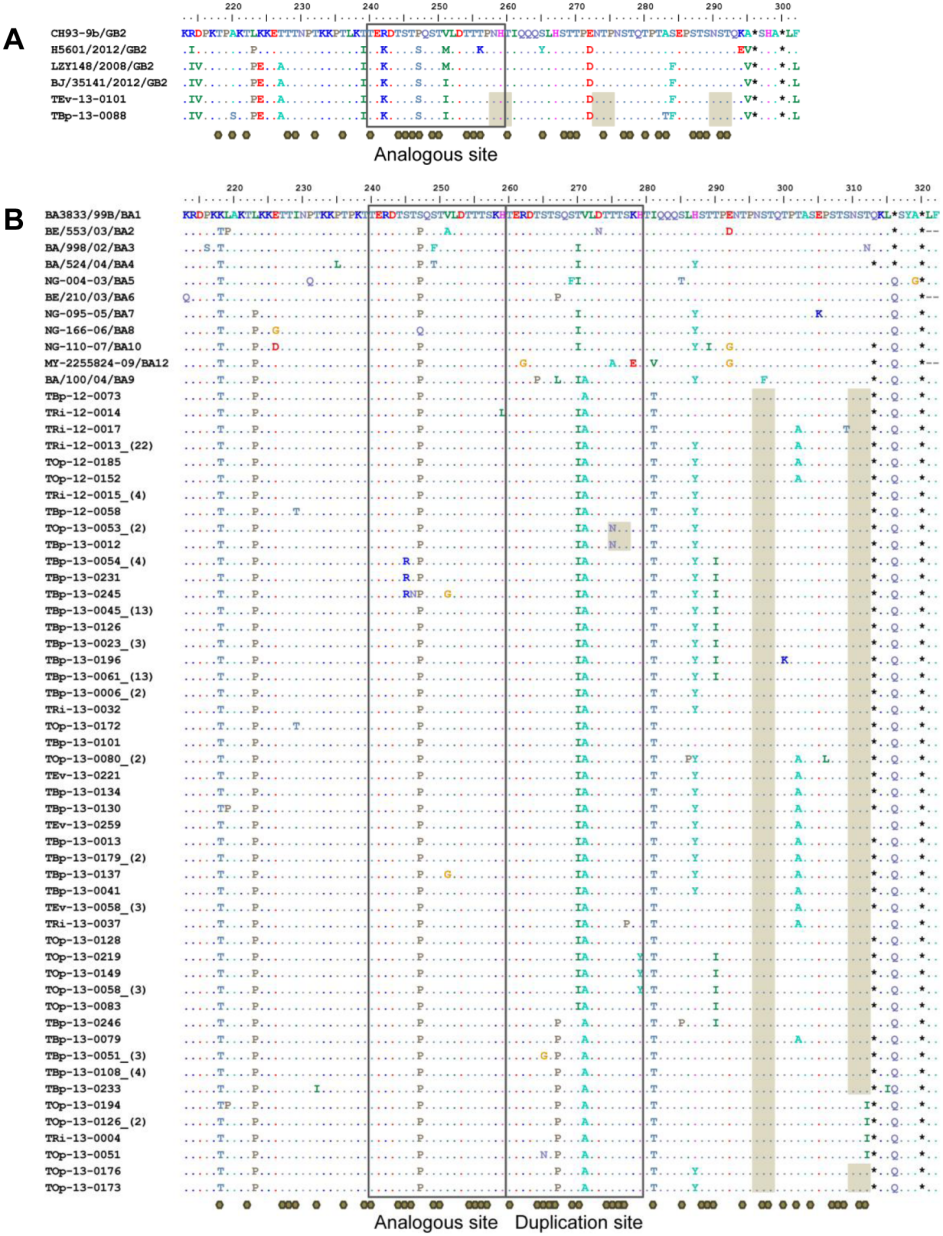
Deduced amino acids of representative HRSV-B G protein in 2012–2013. (A) Two GB2 amino acid sequences corresponding to position 213 to 302 of the 2^nd^ HVR of G protein were aligned with the prototype GB2 strain, CH93-9b (AF065251). (B) Forty-nine representative unique BA9 amino acid sequences corresponding to position 213 to 322 of the 2nd HVR of G protein were aligned with the prototype BA strain, BA3833/99B (AY333362) and the other prototypes of BA branches. Gray shading for predicted N-linked sites, gray octagons for predicted O-linked sites, dots for identical residues, asterisks for stop codon positions, star for positive selection position, and boxes for analogous sites/duplication sites are indicated.

Two GB2 strains were detected in two study sites, EVRMC in Tacloban City of Leyte Island (TEv-13-0101) and BPH in Naval City of Biliran Island (TBp-13-0088) (Fig 4A). The predicted N-linked and O-linked glycosylation sites were found in 3 and 35 positions, respectively. All of the Philippine HRSV-B BA strains had a premature stop codon at amino acid position 313, except for three strains with a stop codon at position 320 (Fig 4B). Previously reported amino acid substitutions such as K218T, L223P, S247P and I281T, which are located outside of the 20-amino acid duplication sites, and S267P, T270I and V271A substitutions, which are found within the duplication sites, were also observed in almost all of the 2012–2013 strains [6, 37–39]. The amino acid change H287Y was found in 73% (85/117) of BA strains. In addition, the substitution T290I was found in one-third (38%, 44/117) of BA strains while the substitution T302A was found in another one-third (35%, 41/117) of the strains. Some BA strains contain the Ser to Pro substitution at position 267 in the duplication site located exactly in the same position (S247P) as in the analogous site. The predicted N-linked and O-linked glycosylation sites were 3 and 46 positions, respectively, which also included the predicted O-linked sugars found in the duplication site. The amino acid mutation T275N within the duplicated site led to a gain of glycosylation site while the T312I substitution led to a loss of glycosylation site.

**Fig 5:**
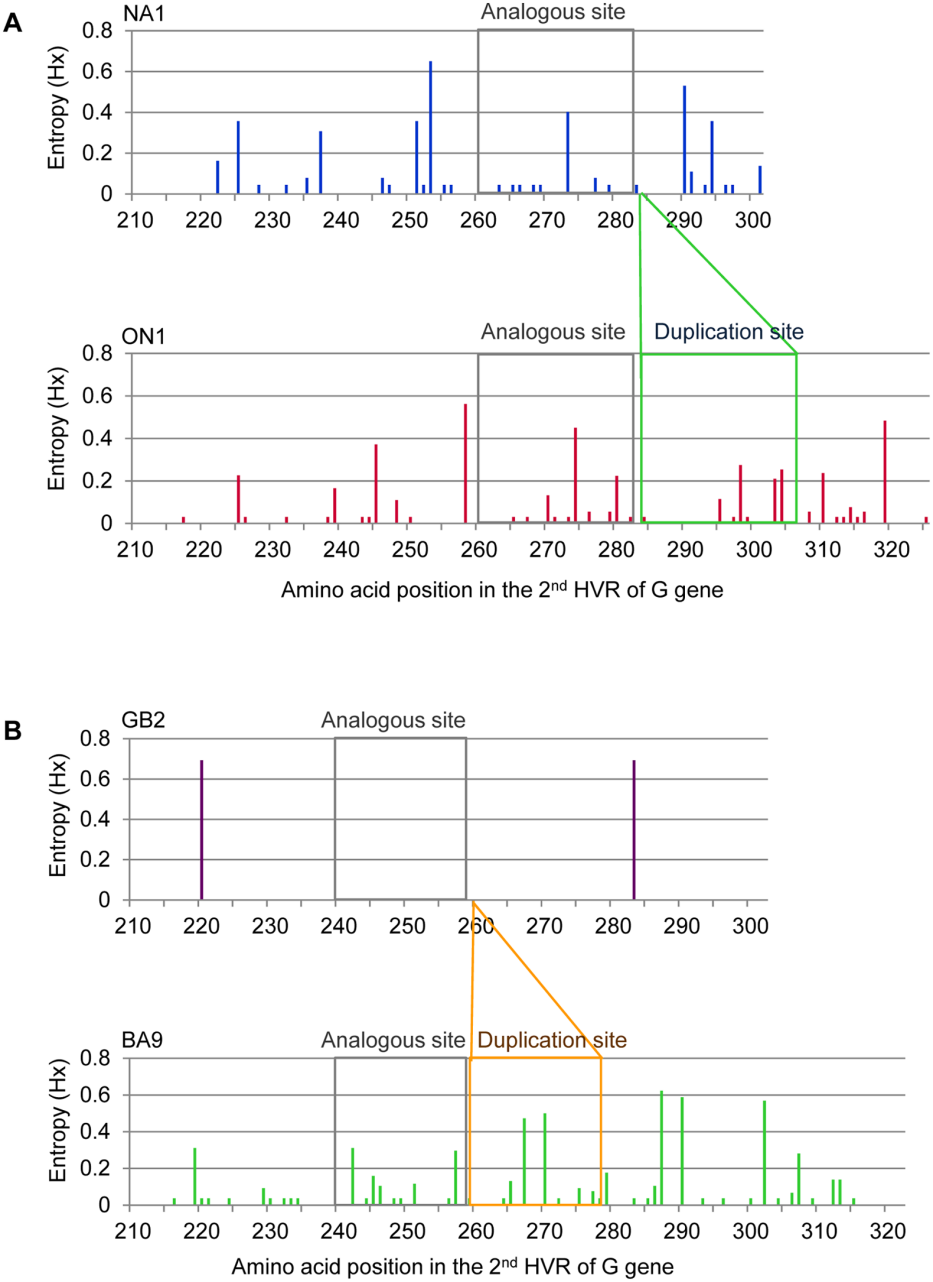
Shannon entropy plots of deduced amino acid sequences of the 2^nd^ HVR of G protein of Philippine strains detected in 2012–2013. Entropy analysis of (A) HRSV-A: NA1 genotype, n = 101 (upper) and ON1 genotype, n = 204 (lower) and (B) HRSV-B: GB2 genotype, n = 2 (upper) and BA9 genotype, n = 116 (lower) in four study sites in the Philippines. The amino acid variability across the second hypervariable region of the G gene is represented by the entropy plot as determined by BioEdit software. The threshold value was set at 0.2. Amino acid sites with entropy values <0.2 are considered conserved and values >0.2 are considered variable.

Shannon entropy analysis of the 2^nd^ HVR of the G protein of Philippine HRSV-A showed that variable sites are located within and outside of the duplicated regions (Fig 5A). Five amino acid positions (positions 274, 280, 298, 303, and 304) in both analogous and duplication sites of ON1 strains were variable. Variable sites of HRSV-B are also distributed within and outside the duplicated regions (Fig 5B). Four amino acid positions (positions 242, 257, 267, and 270) in both analogous and duplication sites of BA9 strains were variable.

### Phylodynamics of HRSV in the Philippines

**Fig 6:**
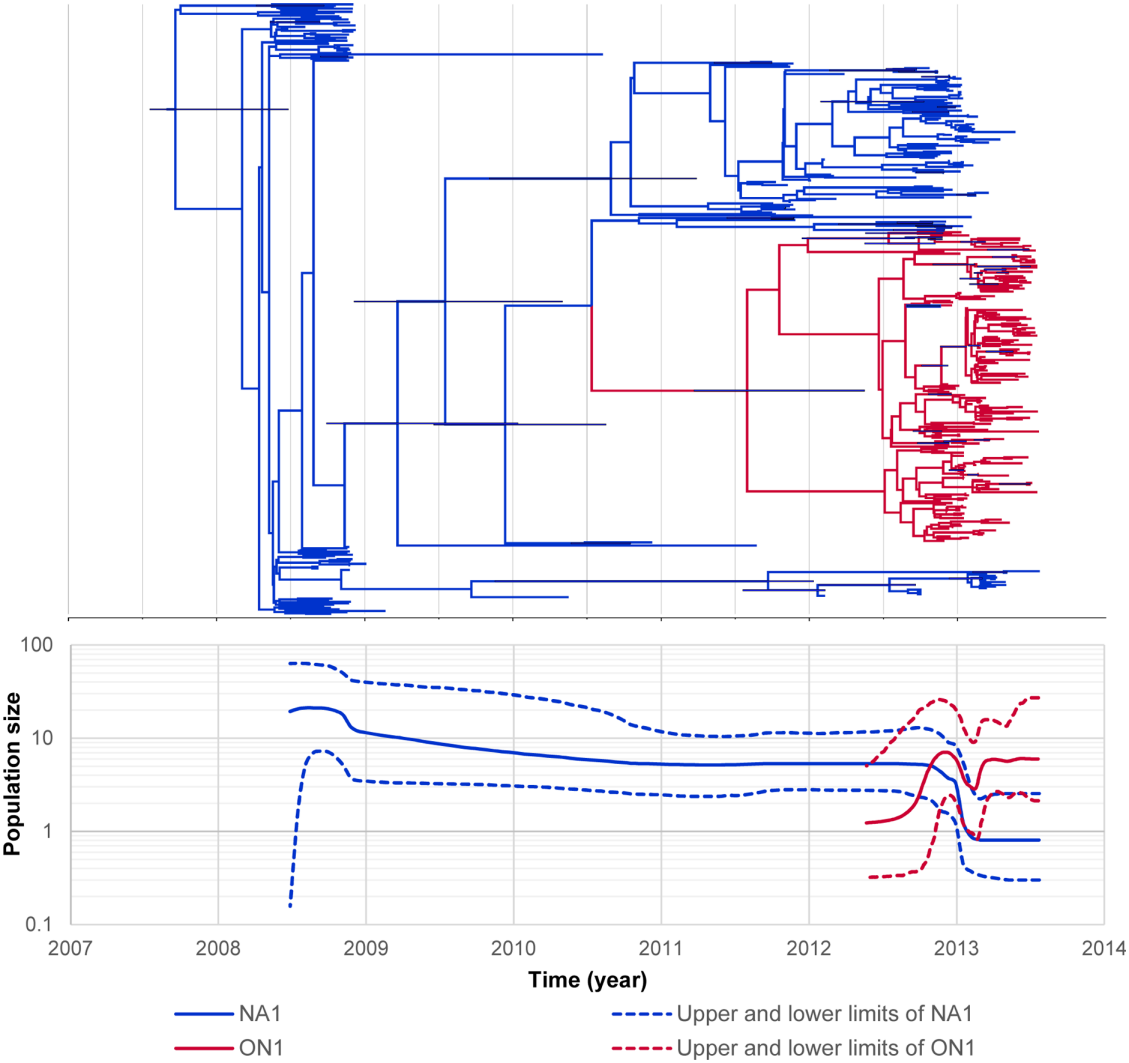
Phylodynamic analysis of HRSV-A from 2008 to 2013. Dated phylogeny (upper) and Bayesian skyline plot (lower) of the NA1 and ON1 genotypes. The NA1 genotype was predominant and its population size showed a decreasing trend from 2009 to late 2012 and then exhibited a sharp decline in 2013. The ON1 genotype emerged around late 2012 and became the predominant genotype with an increasing trend in population size in 2013.

Markov Chain Monte Carlo (MCMC) analysis was performed for all nucleotide sequences of HRSVs detected in the Philippines from May 2008 to April 2012 from our previous report [7] and HRSV sequences from viruses collected from June 2012 to July 2013 in the present study. Dated phylogeny analysis of ON1 genotype (204 sequences) and NA1 genotype (198 sequences) viruses collected during May 2008 –July 2013 showed that the ON1 genotype HRSVs might have emerged in May 2010 (95% HPD, July 2007 to May 2012), while the NA1 genotype HRSVs might have emerged in February 2008 (95% HPD, April 2007 to June 2008) (Fig 6). The NA1 genotype viruses had been circulating since 2008, and its population slightly decreased in late 2012, suddenly decreased in early 2013, and then was steady until the middle of 2013. In contrast, the ON1 genotype viruses emerged in late 2012, suddenly increased in early 2013, and became the predominant genotype of HRSV-A in a short period (October 2012 –July 2013) with a higher evolutionary rate [6.30 x 10^−3^ substitutions/site/year (95% HPD, 3.63 x 10^−3^ to 9.37 x 10^−3^)] than the NA1 genotype [5.40 x 10^−3^ substitutions/site/year (95% HPD, 3.30 x 10^−3^ to 7.74 x 10^−3^)].

**Fig 7:**
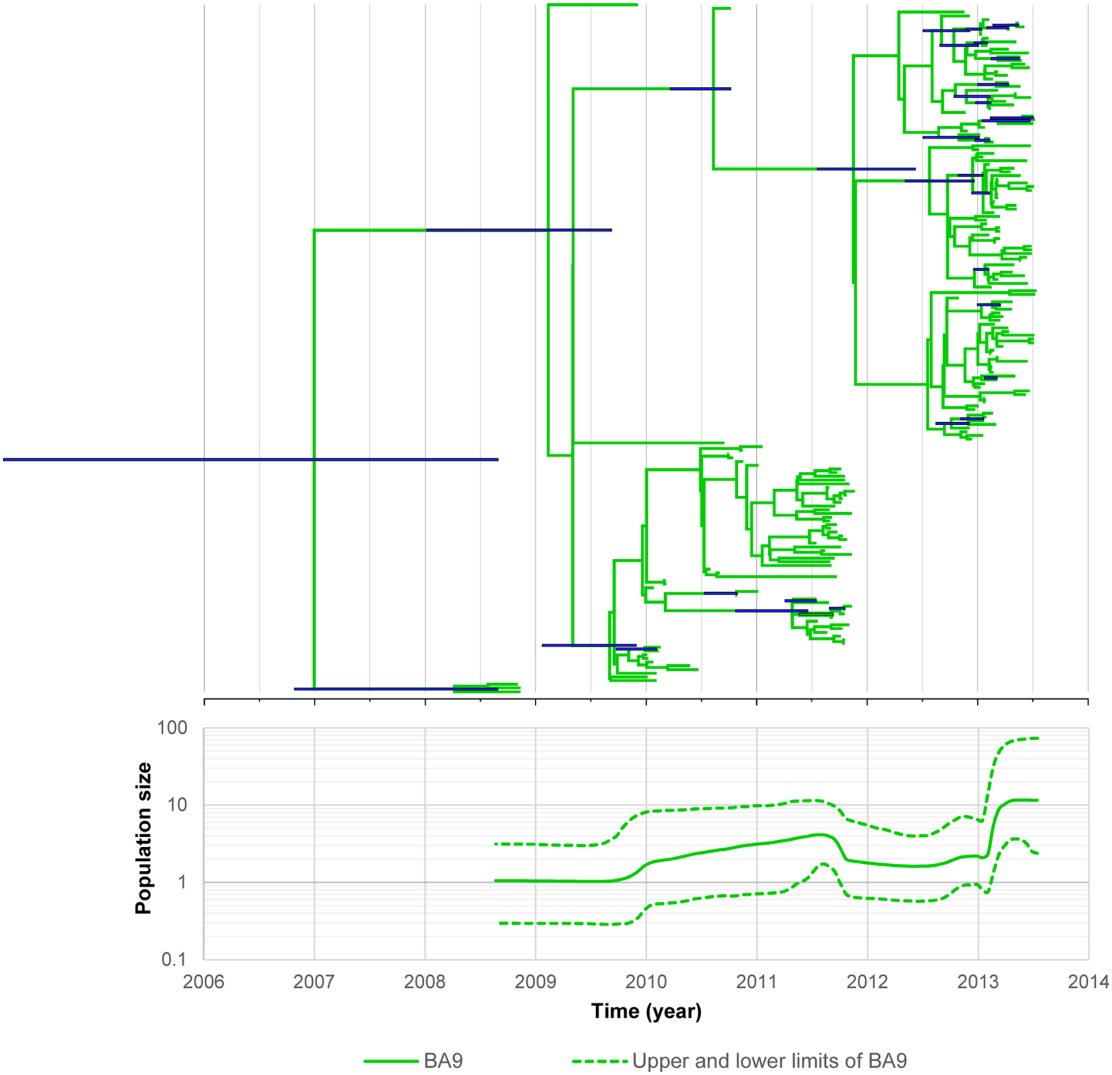
Phylodynamic analysis of HRSV-B from 2008 to 2013. Dated phylogeny (upper) and Bayesian skyline plot (lower) of the BA9 genotype. The population size of the BA9 genotype showed an increasing trend from 2009 and a sharp increase in 2013.

Dated phylogeny analysis of BA9 genotype viruses (186 sequences) detected during May 2008 –July 2013 showed that BA9 genotype HRSVs might have emerged in September 2006 (95% HPD, July 2003 to September 2008) (Fig 7). The BA9 genotype viruses were circulating from 2008 to 2013 then suddenly increased in number in early 2013, with an evolutionary rate of 9.62 x10^-3^ substitutions/site/year (95% HPD, 6.04 x 10^−3^ to 1.36 x 10^−2^). Coalescent analysis was not possible for the GB2 genotype because there were only two strains detected.

### Phylogenetic analysis of GB2 strains detected in the Philippines

**Fig 8:**
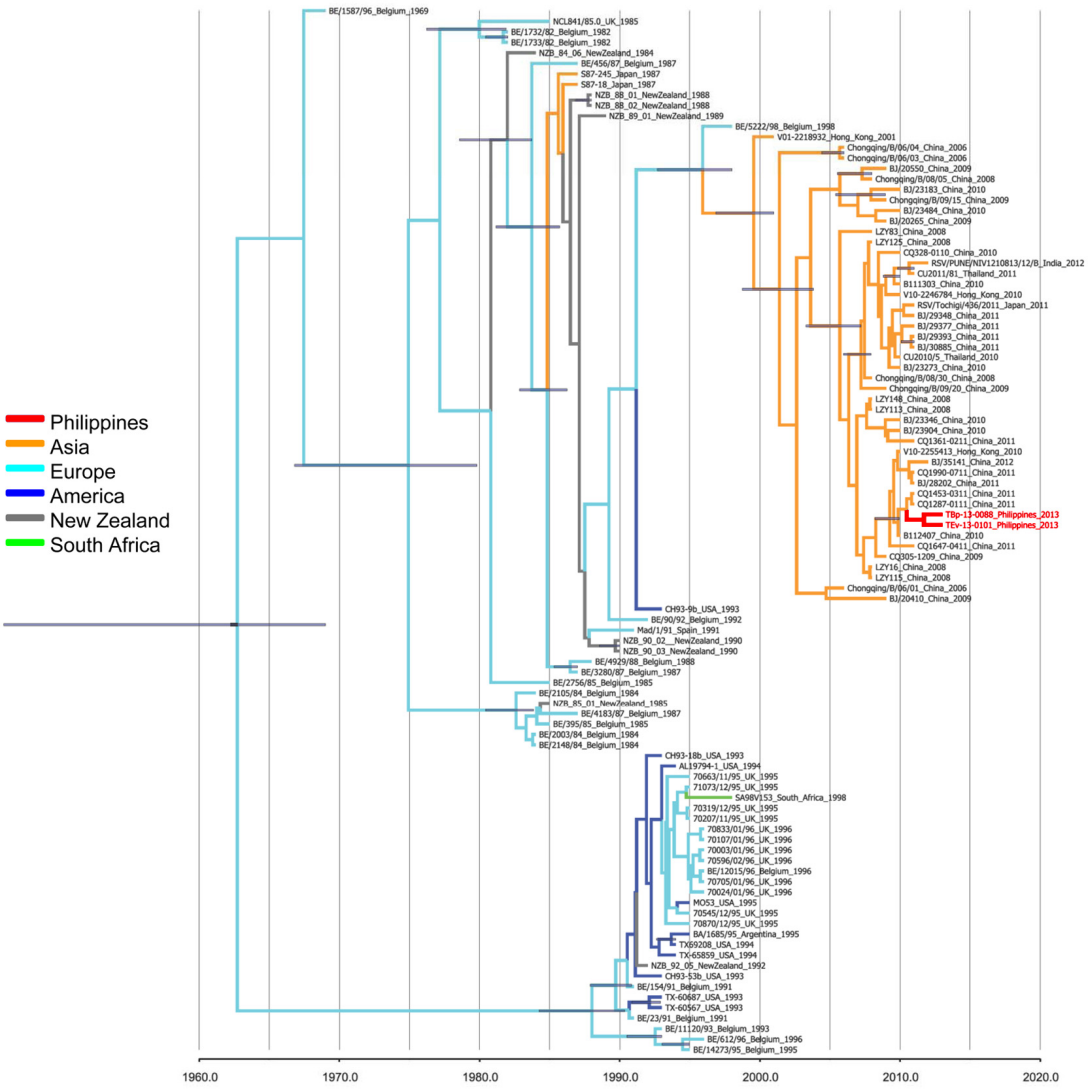
Phylogenetic analysis of GB2 genotype from the Philippines. The phylogenetic tree was constructed by adding 98 strains from other countries that were identified by BLASTn search. The Philippine GB2 strains may share a common ancestor with strains from Asia.

Two GB2 strains that were detected in February 2013, TEv-13-0101 and TBp-13-0088, were analyzed with 97 GenBank strains with known collection dates obtained from a BLASTn result as of May 22, 2014 and a reference strain known as GB2 strain (BE/90/92: AY751247). GB2 genotype HRSVs in the Philippines might have emerged in 2011 and are closely related with strains detected in several locations in Asia, including China, Hong Kong, Thailand, India, and Japan (Fig 8).

Root-to-tip linear regression analysis of HRSV–A and–B sequence data from 2012–2013 by Path-O-Gen software revealed no strong temporal signal in both subgroups with correlation coefficient (*r*) of -0.071 for HRSV-A and 0.089 for HRSV-B (S1 and S2 Figs). When the dataset were grouped according to genotypes, higher *r-*values were obtained with *r* = 0.429 for NA1 and 0.315 for ON1 but no temporal signal for BA9 with r = -0.3722 (S3–S5 Figs)

## Discussion

HRSV is a major cause of hospitalizations among children with childhood pneumonia. Globally, 22% of children with ALRI were infected with HRSV [1]. From 2008 to 2012, an etiological study on acute respiratory infection in the Philippines showed that HRSV (19.3%) was one of the most common virus detected from hospitalized children with severe pneumonia [7]. In 2012–2013, HRSV (28.1%) was also shown to be a common cause of ALRI in the present study.

Usually, both HRSV subgroups are cocirculating during each epidemic season but one of the HRSV subgroups predominates [40, 41]. In our previous study in the Philippines, HRSV-A and HRSV-B cocirculated from May 2008 to April 2012. HRSV-A NA1 was the predominant genotype with RSV-B BA9 strains concurrently circulating [7]. In this study, HRSV-A and HRSV-B cocirculation was also observed. More importantly, we identified the emergence of the novel ON1 strain in the Philippines, which became the predominant genotype of HRSV-A, and also the reemergence of GB2, which is a non-duplication type of HRSV-B in 2012–2013.

Novel viruses or novel antigenic variants of a virus can spread rapidly and become the dominant strain in succeeding epidemics. They may also cause larger outbreaks due to the lack of immunity in susceptible populations. The NA1 genotype, a variant of GA2 genotype [42], caused a large outbreak after the first detection in the 2004/05 season in Niigata, Japan [5]. TMRCA (Time to Most Recent Common Ancestor) analysis in this study determined that NA1 emerged in December 2007 in the Philippines. NA1 was found in different parts of the world including India [43], Thailand [23], China [44], and Germany [45], and became the predominant genotype in 2008–2009 in the Philippines [7], 2009–2010 and 2010–2011 in Latvia [15], and 2011–2012 in Pakistan [37].

The novel ON1 genotype, which has a 72-nucleotide duplication in the 2^nd^ HVR of the G gene, was first detected in Ontario, Canada in 2010 [4]. In 2011, it was detected in other countries such as South Korea, Thailand, Malaysia, and India [8, 20, 23, 43]. In 2012, the ON1 genotype HRSVs had spread further to Japan, South Africa, China, Kenya, Germany, Latvia, Italy, Croatia, and Cyprus [14–18, 24, 39, 46, 47]. The ON1 genotype became the predominant genotype in several countries including South Korea, Kenya, Cyprus, and Italy [18, 20, 46, 47]. In the Philippines, the ON1 genotype was first detected in October 2012 (Table 1, S6 Fig) and it became the dominant genotype in 2012–2013. Within only one year, ON1 emerged and became the predominant genotype of HRSV. The ON1 virus may have been introduced once into the country and subsequently spread to different areas as evidenced by monophyly of Philippine ON1 strains with nearest relatives from other countries.

**Table 1:** Dates of emergence of ON1 strains in the world.

Year	Month	Country	Place	Strain name	Accession No.	Ref.
2010	Nov	Canada	Ontario	ON9-1110A	-	[4]
2011	Aug	Thailand	Bangkok	CU2011/91	KC342406	[23]
		South Korea^a^	-	-	-	[20]
	Sep	India	Pune	RSV-A/NIV1114046/11	KC731482	[22, 43]
	Nov	Malaysia	Kuala Lumpur	MY-2444006-11	JX256871	[8]
2012	Jan	Japan	Chiba	Chiba-C/24031	AB698559	[24]
		Italy^a^	Ancona; Roma	02253AN/2012; 0284RM/2012	JX988448; JX988452	[46]
	Feb	South Africa	Cape Town	RXH/ON1/002	JX885731	[16]
		Germany	Bavaria	WUE/5577/12	JX912358	[14]
		China	Beijing	BJ/35320	KC559440	[21]
		Kenya^a^	Mombasa	KEN/Kilifi/111395/13-Feb-2012	KF587911	[13]
	Mar	Latvia	Riga	LV/029/12	KF030154	[15]
	May	Peru	Lima	RSVA/Homo_sapiens/PER/IPE01270/2012	KJ627264	GenBank
	Oct	Philippines^a^	Palawan	TOp-12-0117	KM873424	This study
	Nov	USA	Tennessee	RSVAHomo sapiens/USA/LA2_07/2012	KJ672468	GenBank
	Dec	Cyprus^a^	Nicosia	-	-	[47]
		Croatia	-	HR3445-12	KF057865	GenBank

The first ON1 strains detected in each country were identified by the collection dates in GenBank or in published papers. The prototype strain was detected in November 2010 in Ontario, Canada and then detected in other countries.

^a^ Countries in which the novel ON1 was a predominant genotype of HRSV-A as of 10^th^ October, 2014

The genotype ON1 in this study was estimated to have emerged around May 2010 as determined by TMRCA analysis, and the total number of positive cases was higher than the genotype NA1 in 2012–2013. Similarly, the estimated emergence date of the global ON1 strains was late 2010 [44]. In contrast, a report from Japan suggested that genotype ON1 emerged in 2001 by analyzing with ON1 strains obtained from Canada, South Korea, Italy, Germany, South Africa, and Japan [24] and another published paper showed that the genotype ON1 might have emerged in 2005 by analyzing with strains obtained from Canada, South Korea, Italy, Germany, South Africa, Japan, Croatia, Thailand, and China, as well as the Philippines [48]. These differences on the estimated time of emergence of genotype ON1 might be due to variation in the selection of strains in each analysis.

The first BA genotype of HRSV-B with a 60-nucleotide duplication in the G gene was detected in Argentina in 1999 and spread throughout the world [12]. It became the predominant genotype in many countries including in 2001–2002 and 2004–2005 in Belgium [49], and in 2002–2003 in Buenos Aires, Argentina [36] and Niigata, Japan [50]. BA9, a subgenotype of HRSV-B BA was first described in Niigata, Japan [6]. After that, some countries including Vietnam [51], India [22, 43], China [21, 39], Croatia [52], and Thailand [23] reported BA9 as the predominant genotype among HRSV-B viruses. Some countries including Malaysia [8], Latvia [15], and Saudi Arabia [53] reported other BA genotypes as the predominant genotype, possibly indicating that this genotype was circulating globally. From the TMRCA analysis (Fig 7), the BA9 genotype viruses likely circulated before 2008, when the surveillance study in the Philippines had commenced.

Genetic diversity of HRSV is generated through several mechanisms including nucleotide replacements, usage of alternative stop codons resulting in varying protein lengths, and short sequence insertions, deletions and duplications. The HRSV-A strain Mad/4/91 contains six nucleotide duplication in the G gene [54] and the HRSV-B strain 1355 has three nucleotide duplication in the G gene compared with other strains found at that time [55]. Recently, large nucleotide duplications in the G gene have been observed; 60 nucleotides in HRSV-B and 72 nucleotides in HRSV-A. The mechanism of duplication is still unknown and many researches attempted to answer this question. One theory is “backtracking” of RNA-dependent RNA polymerase induced by a stable loop of RNA genome [4, 56].

Variable amino acid sites within and outside the duplicated regions of the 2^nd^ HVR of G protein were identified using Shannon entropy analysis. The entropy values obtained for this region of the HRSV (range 0–0.8, threshold = 0.2) are higher than the entropy values of the hemagglutinin of A (H3N2) influenza viruses (range 0–0.4, threshold = 0.1) [57]. The positively-selected amino acid position 274 in ON1 strains has a high entropy value which suggests that the amino acid in this position is prone to be substituted.

More than half (54.4%) of the ON1 strains had amino acid changes in the second hypervariable region of the G protein and had diversified from the prototype Canadian strain. The remaining ON1 strains (45.6%) were identical in amino acid sequence to the Canadian ON1 strain. However, mutations occurring in nucleotide sequences that do not result in a change in the amino acid sequences were found in this group (data not shown). These silent mutations include an A to G change at codon position 222 (1.13%), C to T at codon position 226 (1.13%), T to C at codon position 239 (55.68%), C to T at codon position 258 (2.27%), G to A at codon position 262 (1.13%), and A to C change at codon position 277 (1.13%).

There is an accumulation of amino acid changes over time observed in the molecular evolution of HRSVs. The longer the period that HRSVs have circulated, the more diversified their G-gene sequences have become [12]. Previous reports showed that the evolutionary rate of HRSV-B (7.76 x 10^−4^ to 5.89 x 10^−3^ substitutions/site/year) is faster than HRSV-A (6.47 x 10^−4^ to 4.68 x 10^−3^ substitutions/site/year) [17, 58, 59]. Similarly, our findings showed that the evolutionary rate of BA9 was the fastest followed by ON1 and NA1. However, the absence of strong temporal signal for the BA9 dataset makes this estimate not reliable. Possible reasons for the weak signal of molecular clock-like structure might be due to limited sampling period in our study and sampling of similar sequences in the same period [60, 61]. Although NA1 viruses have been circulating in the Philippines longer than ON1 viruses, NA1 viruses have a lower evolutionary rate. The mean substitution rates for Philippine NA1 and ON1 strains are comparable in magnitude (x10^-3^) and overlapping 95% HPD with published data [24, 62]. Furthermore, a Lys (L) to Pro (P) change (16.7%) at the positively-selected amino acid position 274 of Philippine ON1 strains was also frequently found in strains from Italy [46], Kenya [18], and Germany [45].

From the alignment of deduced amino acid (aa) sequences, premature stop codons were found generating shorter open reading frames (ORFs) [4] such as the stop codon position change from aa299 to aa298 for NA1 viruses and stop codon position change from aa323 to aa322 for ON1 viruses compared with the HRSV-A prototype strain A2 (M74568). However, when GB2 and BA9 sequences were compared with the HRSV-B prototype strain SW/8/60 (M73545), no premature stop codons were observed (alignment with prototype strain not shown). Virus protein glycosylation impart various advantages to virus survival and virulence, and immune evasion. During viral evolution, glycosylation sites are easily added and deleted resulting in viral glycoprotein diversity. The second hypervariable region of the attachment G glycoprotein has a mucin-like structure containing a high amount of serine, threonine, and proline. Massive O-glycosylation was found, and also N-glycosylation sites that were reported previously [4, 43] except for a loss of N-glycosylation at amino acid positions 254 (T254K) and 278 (N278K) in the duplication sites of ON1 and BA9 strains, respectively. Taken together, the acquisition of new amino acid substitutions in the G protein that contribute to HRSV genetic variability and diversity, large gene duplication events, and the loss and gain of glycosylation possibly support the continued circulation of HRSVs by virus escape from host antibodies and cytotoxic T cells (CD8) [63].

Two strains of GB2 genotype were detected in February 2013. GB2 is one of the genotypes of HRSV-B that has no 60-nucleotide duplication in the 2^nd^ HVR of the G gene. The first GB2 virus was detected in Belgium in 1987 [10] indicating that genotype GB2 viruses have been circulating for more than 20 years. In Asia, GB2 genotype viruses have been circulating for more than 10 years, especially in China, albeit at low incidence rates with only a few samples detected in India and Thailand [23, 43]. GB2 viruses may have some mechanism to survive, or may be endemic somewhere in the world where no sequence data is available. The nucleotide sequences of GB2 viruses available in GenBank are also limited. The BLASTn algorithm was used for finding nucleotide sequences that are closely related to the GB2 viruses from the Philippines in order to define its nearest relatives. Phylogenetic analysis showed that Philippine GB2 strains are most closely related to strains from China.

The HRSV-B BA genotype viruses have been circulating for more than 10 years and within a decade it has replaced other circulating subgroup B viruses, particularly those without the 60-nucleotide duplication, based on published reports [12]. Although in recent years, a reemergence of non-BA viruses, including GB2, have been reported suggesting that the advantage of selection of BA might decrease [13].

In conclusion, we report the emergence of the novel ON1 genotype in the Philippines and its predominance in the period 2012–2013. The ON1 genotype is the second HRSV genotype with large nucleotide duplication in the G gene. It will be interesting to observe whether it will follow the trend of the BA genotype in replacing other same subgroup genotypes. So far, the ON1 genotype has spread around the world within 1 to 2 years of its emergence compared with 3 to 4 years for BA, although that could be attributed to a more active surveillance in several countries at present compared with that 10 years ago. We also report the reemergence of the GB2 genotype in the Philippines, which was first detected more than 20 years ago and reappeared in Asia in recent years.

## Supporting Information

S1 Fig Root-to-tip linear regression of HRSV-A strains circulating from June 2012-July 2013. (PDF)

S2 Fig Root-to-tip linear regression of HRSV-B strains circulating from June 2012-July 2013. (PDF)

S3 Fig Root-to-tip linear regression of NA1 strains circulating from May 2008-July 2013. (PDF)

S4 Fig Root-to-tip linear regression of ON1 strains circulating from October 2012-July 2013. (PDF)

S5 Fig Root-to-tip linear regression of BA9 strains circulating from May 2008-July 2013. (PDF)

S6 Fig Dates of first detection of ON1 strains in each study site. (PDF)

S1 Table Nucleotide sequences of G gene-specific primers used in real-time polymerase chain reaction for HRSV screening. (PDF)
