# Supplementary material for: Molecular Characterization of Human Respiratory Syncytial Virus in the Philippines, 2012-2013
Source: PLoS One. 2015 Nov 5;10(11):e0142192. doi: 10.1371/journal.pone.0142192 (PMC4635013; doi:10.1371/journal.pone.0142192)
Supplement: S3 Fig — (PDF) [file pone.0142192.s003.pdf]

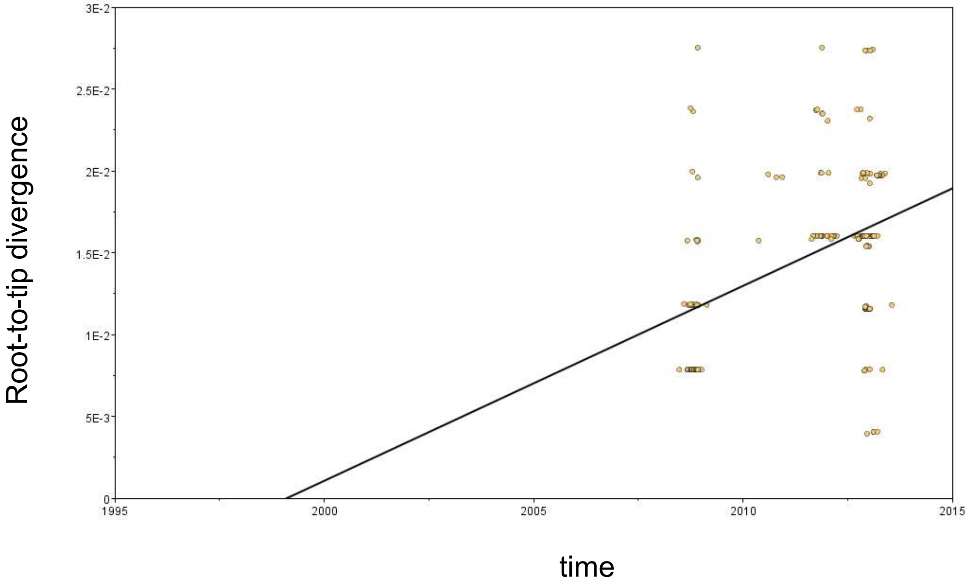

Figure S3. Root-to-tip linear regression of NA1 strains circulating from May 2008 – April 2012. The correlation coefficient (r) value was 0.4296 and the R-squared (r^2) value was 0.1846.
