# Supplementary material for: Molecular Characterization of Human Respiratory Syncytial Virus in the Philippines, 2012-2013
Source: PLoS One. 2015 Nov 5;10(11):e0142192. doi: 10.1371/journal.pone.0142192 (PMC4635013; doi:10.1371/journal.pone.0142192)
Supplement: S5 Fig — (PDF) [file pone.0142192.s005.pdf]

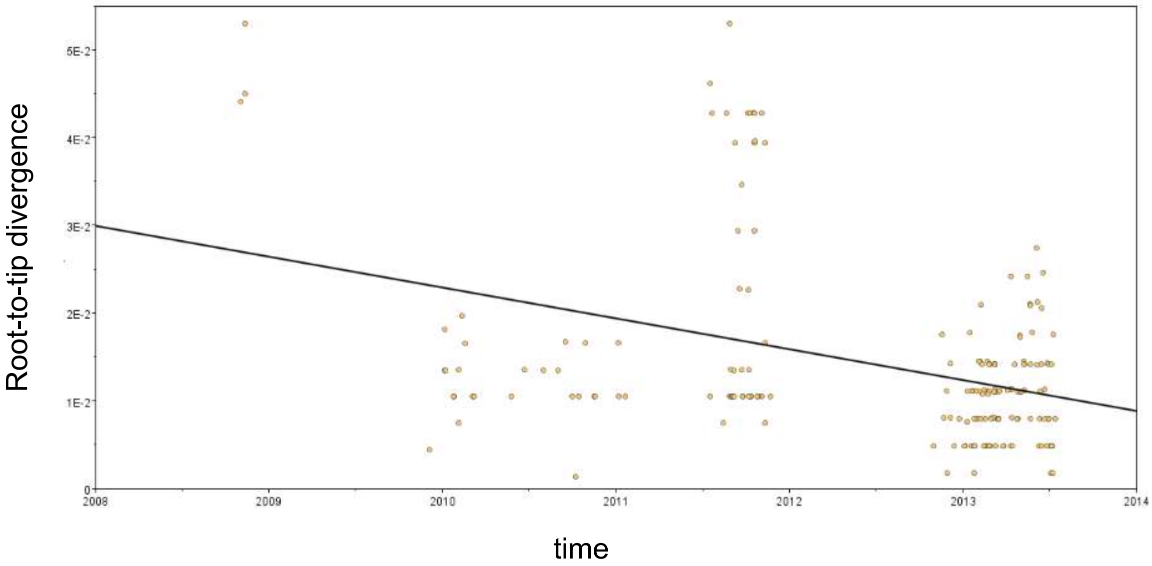

Figure S5. Root-to-tip linear regression of BA9 strains circulating from May 2008 - July 2013.

The r value was -0.3722 and the r^2 value was 0.1386.
