# Supplementary material for: Molecular Characterization of Human Respiratory Syncytial Virus in the Philippines, 2012-2013
Source: PLoS One. 2015 Nov 5;10(11):e0142192. doi: 10.1371/journal.pone.0142192 (PMC4635013; doi:10.1371/journal.pone.0142192)
Supplement: S6 Fig — (PDF) [file pone.0142192.s006.pdf]

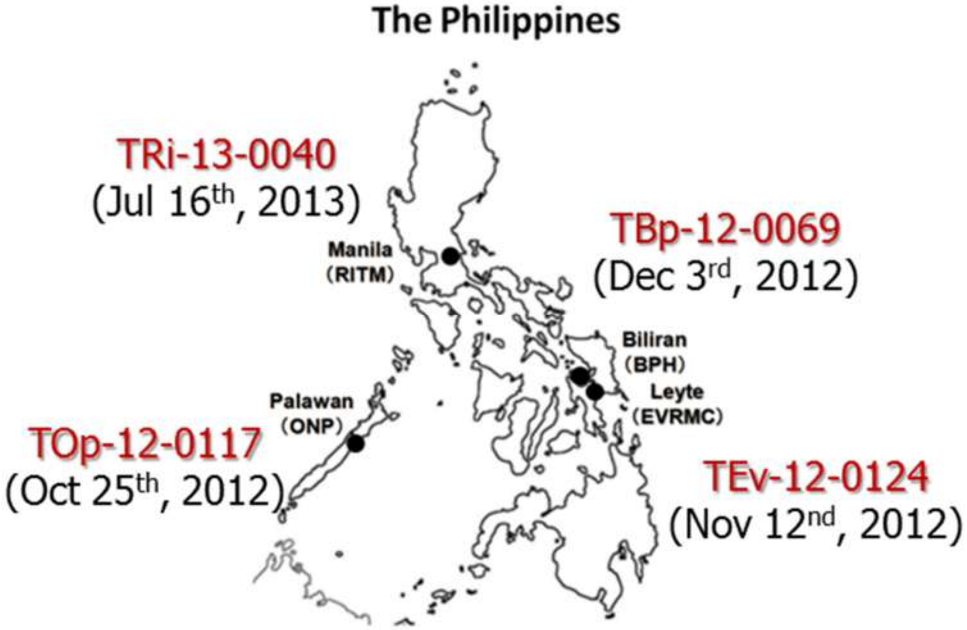

Figure S6. Dates of first detection of ON1 strains in each study site. BPH in Naval City of Biliran Island, EVRMC in Tacloban City of Leyte Island, ONP in Puerto Princesa City of Palawan Island, and RITM in Metro Manila in 2012-2013
