# Supplementary material for: Molecular Characterization of Human Respiratory Syncytial Virus in the Philippines, 2012-2013
Source: PLoS One. 2015 Nov 5;10(11):e0142192. doi: 10.1371/journal.pone.0142192 (PMC4635013; doi:10.1371/journal.pone.0142192)
Supplement: S1 Table — (PDF) [file pone.0142192.s007.pdf]

Table S1. Nucleotide sequences of G gene-specific primers used in real-time polymerase chain reaction for HRSV screening.

Primer name	Sequence (5'→3')	Position
Forward	GCTCTTAGCAAAGTCAAGTTRAATGATACA	1143-1172
Reverse[a]	GTTTYTGCACATCATAATTRGGAGT	1266-1242
Probe[b]	VIC-CTRTCATCCAGCAAATAYACYATCCAACGKAGYACAGG-MGB	1191-1228

Nucleotide position of primer annealing site based on HRSVA strain A2 (M74568)

[a, b] modified from Bonroy C, Vankeerberghen A, Boel A, De Beenhouwer H. Use of a multiplex real-time PCR to study the incidence of human metapneumovirus and human respiratory syncytial virus infections during two winter seasons in a Belgian paediatric hospital. Clin Microbiol Infect. 2007; 13: 504-9.
